# Disabilities and Activities of Daily Living Among Veterans With Old Hip Disarticulation and Transpelvic Amputation

**DOI:** 10.5812/atr.16003

**Published:** 2014-03-25

**Authors:** Amir Reza Kachooei, Mohamad Hosein Ebrahimzadeh, Mohamad Hallaj Moghadam, Asieh-sadat Fattahi, Shiva Razi, Maryam Salehi, Hasan Azema

**Affiliations:** 1 Department of Orthopedic Surgery, Orthopedic Research Center, Faculty of Medicine, Mashhad University of Medical Sciences, Mashhad, IR Iran; 2 Endoscopic and Minimally Invasive Surgery Research Center, Department of Surgery, Faculty of Medicine, Mashhad University of Medical Sciences, Mashhad, IR Iran; 3 Community Medicine Department, Faculty of Medicine, Mashhad University of Medical Sciences, Mashhad, IR Iran; 4 Janbazan Medical and Engineering Research Center, Tehran, IR Iran

**Keywords:** Activities of Daily Living, Veterans, Brody Myopathy

## Abstract

**Background:**

The Iran-Iraq imposed war lasted eight years and was one of the longest wars of the last century. Twenty-three years have passed since the war ended, but little has been discussed about the long-term results of war amputations in the literature.

**Objectives:**

In this long-term study, we have evaluated the activities of daily living among veterans with hip or hemipelvis amputations.

**Patients and Methods:**

A cross-sectional study was performed on Iran-Iraq war veterans with hip or hemipelvis amputations in Iran. Eighty-four (96.5%) veterans out of 87 registered veterans with hip or hemipelvis amputations participated in the study. The degree of independence for activities of daily living (ADL) was assessed by the Barthel index. The degree of independence for instrumental activities of daily living (IADL) was assessed by the Lawton-Brody scale.

**Results:**

The average follow-up time was 26.6 ± 3.7 years. The average age of veterans was 44.1±7 years old. Of 84 amputees, 57 (67.85%) had limitations in at least one domain of the ADL. The most common single item that affected the patients was ascending and descending stairs seen in 45 (78.9%) veterans, followed by eating seen in 4 (7.01%) veterans. In addition, 70 (83.33%) had limitations in at least one domain of the IADL. The most common single item that affected the veterans was shopping seen in 56 (80%), followed by responsibility for own medications seen in 13 (18.57%) veterans. Spearman correlation coefficient of the sum scores of ADL and IADL showed an intermediate to strong correlation (r = 0.58).

**Conclusions:**

Increasing dependency in ADL is accompanied by increasing dependency in IADL. In the past, the duty of health care providers was saving the life of veterans due to injuries while at present, because these injuries occurred in young and healthy individuals, the need for increased function is being highlighted.

## 1. Background

The Iran-Iraq imposed war lasted eight years and was one of the longest wars of the last century in which many healthy, young people took part and were left with different injuries. Presently, 20801 veterans suffer from various types of limb amputations and 12981 veterans underwent lower limb amputations (1).

Twenty-three years have passed since the war ended and the negative consequences of the war have affected the private life, health care, and the society of these veterans that also impose an enormous cost on their families and society. Providing efficient amputation management and care for veterans is important; being from the younger generation, they had a significant role in the defense of their country (2-4) and have survived their injuries. Hence, they should be managed properly to return them back to their previous level of function in order to prevent them from becoming a burden on their family for the rest of their lives.

Little has been discussed about the long-term results of the amputations due to war in the literature (4, 5). Knowledge of long-term problems and difficulties in veterans is needed to offer them better assistance and support (6). In this long-term study, we evaluated the quality of life among veterans with hip or hemipelvis amputations. To our knowledge, this is the first study of its kind in the current literature dealing with disabilities and daily activities in amputees with hip and hemipelvis amputations.

## 2. Objectives

In this long-term study, we aimed to evaluate the activities of daily living among veterans with hip or hemipelvis amputations.

## 3. Patients and Methods

A cross-sectional study was conducted on the Iran-Iraq war veterans with hip or hemipelvis amputations who lived in various parts of Iran. Eighty-four (96.5%) veterans out of 87 registered veterans with hip or hemipelvis amputations participated in this study, which was held in Mashhad, Iran, in 2011. The study team included an orthopedic surgeon, psychiatrist, physical medicine and rehabilitation, and prosthetic specialists. All participants signed an informed consent form before taking part in the study.

Basic characteristics and clinical data were collected. The degree of independence for activities of daily living (ADL) and independence for instrumental activities of daily living (IADL) were assessed by the Barthel index and the Lawton-Brody scale, respectively. 

Data were evaluated by an epidemiologist and community medicine specialist and were analyzed by SPSS 12 (SPSS Inc., Chicago, Illinois, USA).

### 3.1. The Activities of Daily Living

The ADL was evaluated by the Barthel Index (7), which consists of 10 items including eating, taking a bath, grooming, dressing, bowel continence, urine continence, toilet use, transfers (bed to chair and back), mobility (on level surfaces), and stair ascend and descend. Each item was subsumed to two or three outcomes, namely "independent", "needs help", and "dependent". Based on a sum score of all items, patients were classified into two groups: "without limitations" in the case of receiving a full sum score (100%) and "with limitations" in the case of a sum score of less than 100%. Domains of ADL show physical self-maintenance.

### 3.2. Instrumental Activities of Daily Living 

IADL score was evaluated by the Lawton-Brody scale (8), which consists of eight items including ability to use the telephone, shopping, food preparation, housekeeping, laundry, travelling via car or public transportation, responsibility for own medications, and ability to handle finances. The average score ranges from zero (completely dependent) to eight (completely independent). The scale was used to assess all eight domains of function for women but only five for men (food preparation, housekeeping, and laundry were excluded). Domains of IADL show the ability of adaptation to the environment and represent functional competence.

## 4. Results

Eighty-four veterans with hip or hemipelvis amputations participated in this survey. The average follow-up time was 26.6 ± 3.7 years ranging from 23 to 32 years. The average age of veterans was 44.1 ± 7 years old with a minimum and maximum age of 36 and 72 years old, respectively. Fifty-nine (70.2%) amputees were single before being injured, but 79 (94%) amputees were married at the time of study. The average number of veterans' children was 3.1 ± 1.7 (Table 1). Forty-five (53.5%) amputees participate in some kind of sport. The most common sports that they participated in were volleyball and basketball, consecutively. Fifteen (17.8%) veterans were on the national Paralympics volleyball and basketball teams, 12 (14.2%) cases had participated in swimming, 11 (13%) cases in mountain climbing, and 7 (8.3%) cases in shooting sports.

**Table 1.** Basic Characteristics of the Study Participants

Characteristics	Values ^a^
**Age, y**	
< 40	10 (12)
40-44	42 (50)
45-49	22 (26)
≥ 50	10 (12)
**Sex**	
Male	81 (96.4)
female	3 (3.5)
**Employment status**	
Employed	62 (73.8)
Unemployed	22 (26.1)
**Injury side**	
Left	36 (42.8)
Right	40 (47.6)
Bilateral	8 (9.5)
**Year of injury**	
< 1981	10 (12)
1981-1985	32 (38)
1986-1989	42 (50)

^a^ Data are presented as No. (%).

### 4.1. Ambulation

The participants had the following ambulatory conditions: normal ambulation without any aid in 4 (4.7%) amputees, abnormal ambulation without any aid in 9 (10.7%), ambulation using a single crutch in 12 (16%), ambulation using double crutches in 50 (59.5%), ambulation using a walker in 1 (1.3%) and mobilization by wheelchair in 7 (9.3%). Among these veterans, 50 (59.5%) patients complained of decreased ambulation velocity in comparison with the previous year.

Among these 84 hip or hemipelvis amputees, 66 (78.5%) veterans were dissatisfied with their prosthesis and 41 (48.8%) veterans abandoned their prosthesis. It was owing to its heaviness in 16 (19%), pain stimulation in 6 (15%), skin disorders in 6 (7.1%), fatigue in 8 (9.5%), need for frequent repair of prosthesis in 7 (17.5%), need for frequent prosthesis adjustment in 5 (12.5%), need for prosthesis replacement in 7 (17.5%), stump ulcer in 2 (2.6%), phantom pain in 1 (1.3%), and other reasons in 8 (20%) patients. Furthermore, an accompanying amputation included finger amputation in 1 (1.1%), below elbow amputation in 2 (2.2%), and above-elbow amputation in 2 (2.2%) patients.

### 4.2. Activities of Daily Living: Single Items and Sum Score

Of 84 amputees in this study, 57 (67.85%) had limitations in at least one domain of the ADL. The most common single item that affected the patients was ascending and descending stairs in 45 (78.9%) veterans, followed by eating in 4 (7.01%) veterans (Table 2).

**Table 2.** Barthel Index to Assess the Activities of Daily Living (n = 84)

Barthel Index (ADL^a^): Activity	Score	Values ^b^
**Eating**		
Unable	0	0 (0)
Needs help cutting, spreading butter, etc., or requires a modified diet	5	7 (8.3)
Independent	10	77 (91.7)
**Taking a bath**		
Dependent	0	18 (21.4)
Independent	5	66 (78.6)
**Grooming**		
Needs help with personal care	0	4 (4.8)
Independent face/hair/teeth/shaving (implements provided)	5	80 (95.2)
**Dressing**		
Dependent	0	1 (1.2)
Needs help, but can do about half without aid	5	12 (14.3)
Independent (including buttons, zips, laces, etc.)	10	71 (84.5)
**Bowel continence**		
Incontinent (or needs to be given enemas)	0	2 (2.4)
Occasional accident	5	3 (3.6)
Continent	10	79 (94)
**Urine continence**		
Incontinent or catheterized and unable to manage alone	0	1 (1.2)
Occasional accident	5	8 (9.5)
Continent	10	75 (89.3)
**Toilet use**		
Dependent	0	0 (0)
Needs some help, but can do something alone.	5	9 (10.7)
Independent (on and off, dressing, wiping)	10	75 (89.3)
**Transfers (bed to chair and back)**		
Unable, no sitting balance	0	0 (0)
major help (one or two people, physical), can sit	5	1 (1.2)
minor help (verbal or physical)	10	11 (13.1)
independent	15	72 (85.7)
**Mobility (on level surfaces)**		
Immobile or < 50 yards	0	0 (0)
Wheelchair independent, including corners, > 50 yards	5	8 (9.5)
Walks with the help of one person (verbal or physical) > 50 yards	10	4 (4.8)
Independent (but may use any aid; for example, cane) > 50 yards	15	72 (85.7)
**Stairs**		
Unable	0	9 (10.7)
Needs help (verbal, physical, carrying aid)	5	36 (42.9)
Independent	10	39 (46.4)

^a^ Abbreviations: ADL, activities of daily living.

^b^ Data are presented as No. (%).

### 4.3. Instrumental Activities of Daily Living: Single Items and Sum Score

In the 84 amputees, 70 (83.33%) had limitations in at least one domain of the IADL. The most common single item that affected the veterans was shopping in 56 (80%), followed by responsibility for own medications in 13 (18.57%) veterans (Table 3). 

**Table 3.** Lawton-Brody Score to Assess the Instrumental Activities of Daily Living (n = 84)

Lawton-Brody Score (IADL^a^)	Values^b^
**Ability to use telephone**	
Dependent	0 (0)
Independent	84 (100)
**Shopping**	
Dependent	56 (66.7)
Independent	28 (33.3)
**Mode of transportation**	
Dependent	20 (23.8)
Independent	64 (76.2)
**Responsibility for own medications**	
Dependent	42 (50)
Independent	42 (50)
**Ability to handle finances**	
Dependent	13 (15.5)
Independent	71 (84.5)

^a^ Abbreviation: IADL, instrumental activities of daily living.

^b^ Data are presented as No. (%).

### 4.4. Correlation Between Activities of Daily Living and Instrumental Activities of Daily Living

We calculated the correlation between the sum score of ADL and IADL. Spearman correlation coefficient of sum scores of ADL and IADL was 0.58 (P = 0.001) that showed an intermediate correlation.

## 5. Discussion

The eight-year Iran-Iraq war, which was initiated by the former Iraqi dictator Saddam-Husain in 1980, left more than 200,000 casualties and more than 400,000 veterans in Iran (9).

A few studies reported clinical outcomes of war-related amputation (10-12), but disabilities and clinical results of lower limb amputations including war-related hip and hemipelvis amputations have not been fully evaluated yet. This kind of injury is rare and life threatening and it needs a multidisciplinary team to manage it and its complications (13).

In our study, different aspects of daily living activities were assessed by the Barthel index and Lawton-Brody scale. The highest dependency among 84 veterans with hip or hemipelvis amputation was in ascending and descending stairs (78.9%), followed by eating (7.01%). Therefore, 85.91% of the patients had limitations in ascending and descending stairs in combination with eating. The results of our study were similar to a study by Roehrig, in which the greatest dependency was in "ascending and descending stairs" and "shopping" (14).

Amini et al. evaluated Iranian blind veterans by the Barthel index (15) and showed that an increase in age and time elapsed since injury were the predicting factors of increasing dependency, as well as lower education level, comorbidities, the influence of accompanying injuries, high blood pressure, diabetes, admission to a hospital during the previous year, and loss of visual and hearing acuity (16, 17). In another Iranian survey on bilateral lower limb amputations among 335 veterans, Ashraf et al. showed that the highest dependency was in the transfer activities (27.8%) and bathing (23.3%) domains, but the highest independency was in the eating domain (97.6%). In his study, upper cervical pain was associated with dependency in bowel, bladder, and dressing domains; moreover, lower cervical pain was associated with dependency in toileting and dressing domains, and pain in the lumbosacral region was associated with the level of amputation as well as dependency in transfer activities and toileting domains. They concluded that vertebral pain in bilateral lower limb amputations had an impact on level of function and therefore, pain management should be a priority (18). In addition, the assessment of the Barthel index showed dependency in at least one domain in American veterans older than 65 years of age (19). In a study on bilateral lower limb amputees in India, there was no significant difference between transtibial and transfemoral amputees while the scores were significantly higher for prosthetic users versus non-prosthetic users. They concluded that prosthesis rehabilitation was more important than the level of amputation with regard to ADL (20).

In the current study, IADL was assessed by the Lawton-Brody scale and due to the high proportion of male veterans, five domains out of eight were assessed. The greatest dependency among these 84 veterans with hip or hemipelvis amputation was shopping (80%) followed by responsibility for own medications (18.57%). Therefore, 98.57% of the patients had limitations in shopping in combination with responsibility for their own medications. According to the current literature, the Lawton-Brody scale was used in studies for other ailments; however, it had not been used for veterans and amputees. Therefore, our study can be used as a basis for future studies.

In our study, the calculation of the correlation coefficient between the sum score of ADL and IADL was 0.58. A correlation coefficient of more than 0.4 is a good correlation, which shows a linear correlation between dependency in ADL and IADL. Hence, increasing dependency in ADL was accompanied by increasing dependency in IADL. Roehrig et al. also achieved the similar results and suggested that an abbreviated form of six items instead of a total of 18 items of the ADL and IADL could be used for screening (14).

Due to the inversion of mortal factors to disabling factors, the average age and the number of senile people in this population is increasing and therefore, the need for rehabilitation services is increasing as well (21, 22). Moreover, rehabilitation protocols aiming to promote the functional abilities and independence of the veterans are needed (23).

In the past, the duty of health care providers was saving the life of veterans due to injuries, but at the present time, because these injuries have occurred in young and healthy individuals, the need for increasing their function is being highlighted (24). Therefore, the aim of war field medical caregivers should be not only saving a life, but also preserving the maximum level of function and independence in the injured veterans, assisting in returning them back to their society (25). This approach begins with proper surgical techniques in the war field and continues with understanding amputees' disabilities as wells as long-term rehabilitation and treatment of sequels and complications.
